# Psychological health, sleep quality, and coping styles to stress facing the COVID-19 in Wuhan, China

**DOI:** 10.1038/s41398-020-00913-3

**Published:** 2020-07-09

**Authors:** Wenning Fu, Chao Wang, Li Zou, Yingying Guo, Zuxun Lu, Shijiao Yan, Jing Mao

**Affiliations:** 1 School of Nursing, Tongji Medical College, Huazhong University of Science and Technology, Wuhan, 430030 China; 2 School of Public Health, Tongji Medical College, Huazhong University of Science and Technology, Wuhan, 430030 China; 3 Department of Neurology, Renmin Hospital of Wuhan University, Wuhan, 430060 China; 4 Children’s Healthcare Department, Tongji Hospital, Tongji Medical College, Huazhong University of Science and Technology, Wuhan, 430030 China; 5 School of public health, Hainan Medical University, Haikou, 571199 China; 6 Research Unit of Island Emergency Medicine, Chinese Academy of Medical Sciences (No. 2019RU013), Hainan Medical University, Haikou, 571199 China

**Keywords:** Scientific community, Psychiatric disorders

## Abstract

To understand Wuhan residents’ psychological reactions to the COVID-19 epidemic and offer a reference point for interventions, an online questionnaire survey was conducted. It included the Disorder 7-Item Scale (GAD-7), the Patient Health Questionnaire 9-Item Scale (PHQ-9), Athens Insomnia Scale, and Simplified Coping Style Questionnaire. Categorical data were reported as numbers and percentages. Multivariate logistic regression models were used to evaluate the association between demographic factors and anxiety, depression, sleep disorder, and passive coping style. A total of 1242 Wuhan residents investigated, 27.5% had anxiety, 29.3% had depression, 30.0% had a sleep disorder, and 29.8% had a passive response to COVID-19. Being female was the risk factor for anxiety (*OR* = 1.62) and sleep disorder (*OR* = 1.36); being married was associated with anxiety (*OR* = 1.75); having a monthly income between 1000 and 5000 CNY (*OR* = 1.44, *OR* = 1.83, *OR* = 2.61) or >5000 CNY (*OR* = 1.47, *OR* = 1.45, *OR* = 2.14) was a risk factor for anxiety, depression, and sleep disorder; not exercising (*OR* = 1.45, *OR* = 1.71, *OR* = 1. 85, *OR* = 1.71) was a common risk factor for anxiety, depression, sleep disorder, and passive coping style; and having a higher education level (bachelor’s degree and above) (*OR* = 1.40) was associated with having a sleep disorder. Wuhan residents’ psychological status and sleep quality were relatively poorer than they were before the COVID-19 epidemic; however, the rate of passive coping to stress was relatively higher.

## Introduction

In December 2019, the novel coronavirus pneumonia broke out in Wuhan, China, and rapidly spread throughout the country. This sudden and unexpected epidemic disrupted the peace of life and dramatically threatened physical and psychological health in China. Due to the high infectivity, mortality rate, and lack of understanding of the virus, the epidemic has caused universal psychological problems. People living in China have been forced to isolate themselves in their homes, and all forms of gathering have been strictly prohibited. People could only engage with the outside world through the internet, and the negative news they found there about the epidemic further aggravated their psychological burden. Mental health problems have seriously deteriorated medical professionals’ performance of tasks involving attention, cognition, and memory and destroyed their confidence, leading to medical errors in the battle against COVID-19^1,2^. Anxiety, depression, and insomnia have significantly impacted the quality of life and physical health of virus-infected patients as well as the general public^3^.

Understanding and guiding the mental health response of the general public in this epidemic promptly may help communities better prepare for emergency public health events^4^. The National Health Commission of China published the National Guideline for Psychological Crisis Intervention for 2019-nCoV^5^, indicating that addressing the mental health repercussions of this epidemic has become a nationwide mission, requiring the attention of the entire society.

However, little information has been gathered about the epidemiology and psychological features of communities until now^1^. We conducted a cross-sectional study to comprehensively describe Wuhan residents’ psychological reactions to the COVID-19 epidemic to offer a reference point for mental health interventions.

## Methods

### Sample and data collection

We conducted a cross-sectional study among residents of Wuhan, China, from February 18, 2020, to February 28, 2020. A total of 1242 valid questionnaires were collected. We selected participants using a convenience sampling technique. The questionnaires were completed through an online survey platform (“SurveyStar,” Changsha Ranxing Science and Technology, Shanghai, China). All participants provided informed consent electronically prior to registration. The informed consent page presented two options (yes/no). Only participants who chose “yes” were taken to the questionnaire page. The inclusion and exclusion criteria of residents are as follows: Inclusion criteria were residents who: (1) aged 18 years and older; (2) living in Wuhan during the outbreaks of COVID-19; (3) have provided informed consent electronically prior to registration. Exclusion criteria were residents who: (1) have been suffering from baseline psychological or sleep-related diseases; (2) having medications for mental or sleep illnesses; (3) offered the questionnaire with logical errors.

To ensure the quality and completeness of the questionnaire results, each mobile phone or computer could only be used once. Logic checks were also built into the background system to identify invalid questionnaires. The answers to all valid questionnaires were automatically entered into a data file and checked by two independent researchers. The survey tools included the Disorder 7-Item Scale (GAD-7), the Patient Health Questionnaire-9 (PHQ-9), Athens Insomnia Scale, and Coping Style Scale.

### Measurement

GAD-7 is gauged with a seven-item scale developed by Spitzer et al.^6^, with a score of 0 to 3 for each item. The total score of the GAD scale ranges from 0 to 21. Those with a total score of 0–4 are regarded as having no anxiety symptoms, those with a total score of 5–9 as having mild anxiety, those with a total score of 10–14 as having moderate anxiety, and those with a total score of ≥15 as having severe anxiety. In this study, those with a total score of equal to or less than 4 were considered as presenting no anxiety symptoms. In contrast, those with a total score of 5 or more were regarded as reporting anxiety symptoms. The GAD-7 scale had good factorial validity and reliability (with Cronbach’s alpha coefficients of 0.901). Furthermore, the validity of the GAD scale in assessing anxiety in Chinese has been confirmed^7^.

The Patient Health Questionnaire (PHQ-9) is a nine-item psychological self-assessment questionnaire for depression-related symptoms^6^. Each item can receive 0–3 points, and the total score of the questionnaire ranges from 0 to 27 points. A total score of 0–4 signals no depressive symptoms, a total score of 5–9 indicates mild depression, a total score of 10–14 indicates moderate depression, a total score of 15–19 means severe depression, and a total score of 20–27 denotes extremely severe depression. The Chinese version of the PHQ-9 has been standardized and corrected. The PHQ-9 had satisfactory reliability (with Cronbach’s alpha coefficients of 0.869) and extensive sensitivity and specificity, which have been validated^8^.

The Athens Insomnia Scale (AIS) is an internationally recognized and generally applicable sleep quality self-assessment for sleep disorders^9^. There are eight items on the scale, and each item is divided into four levels with a 4-point Likert scale. The scores range from 0 to 3: 0 = no problem, 1 = mild delay, 2 = significant delay, 3 = severe delay or no sleep. If the total score for the entire scale is less than 4, it indicates that there is no sleep disorder; if the total score is 4–6, it indicates that the respondent may be developing insomnia; if the total score is more than 6 points, it indicates insomnia. In this study, those with a total score of 0 to 4 were considered as having no insomnia symptoms, while those with a total score of 5 or more were regarded as having insomnia. The Cronbach’s alpha coefficient of the AIS was 0.797, suggesting that its internal consistency was appropriate.

Coping style was measured using the Simplified Coping Style Questionnaire (SCSQ). This questionnaire was developed by Xie et al.^10^ based on the Ways of Coping questionnaire by Folkman and Lazarus^11^. It is a 20-item self-report questionnaire that includes two dimensions. The active coping dimension, consisting of items 1–12, reflects the characteristics of an individual’s active coping style when encountering stress, and the passive coping dimension contains items from 13–20 and refers to the characteristics of an individual’s passive coping style. This questionnaire asks respondents to agree or disagree on a 4-point Likert scale according to how frequently they adopt each item from 0 “never” to 3 “very often,” with higher scores representing greater active/passive coping. In this study, we used the differential value between the standard score of active coping minus the standard score of passive coping to determine the tendency of individual coping styles. Among them, the standard score was yielded by Z-transforming the mean and standard deviation of the positive and negative coping styles. If the differential value was greater than 0, it indicated that the individual generally adopted a positive coping style, and vice versa. This instrument is commonly used in Chinese, and the Cronbach’s alpha coefficients for the two dimensions were 0.916 and 0.808, respectively^12^.

### Data analysis

Data analyses were performed using the SPSS software (Version 22 for Windows, SPSS Inc, Chicago, IL, U.S.A.). Descriptive analysis was made on the basic characteristics of residents by frequency and composition ratio, and compared their differences between basic situation and mental health statuses among various demographics by *χ*^2^ tests. The data were tested for normal distribution. A value of *P* < 0.05 (two-tailed) was considered statistically significant. Multivariate logistic regression models were used to evaluate the association between different factors and anxiety, depression, sleep disorder and passive coping style.

## Results

Table 1 shows the respondents’ demographic characteristics and the differences in their anxiety and depression statuses. Of the 1242 residents remaining in the final analysis, 376 (30.27%) were men, and 866 (69.73%) were women. Of them, 27.5% had anxiety, and 29.3% had depression. There were significant differences in the residents’ anxiety levels based on gender, age, marital status, place of residence, monthly income, frequency of online video communication, and exercise (*P* < 0.05). Female residents reported a higher anxiety level than did men (29.7% vs. 22.4%). The proportion of anxious residents ≤30 years old was lower than that of residents >30 years old (24.5% vs. 35.8%). Married residents were more likely to have anxiety than were single adults (37.0% vs. 22.7%). The proportion of urban residents who showed anxiety symptoms was higher than that of rural residents (30.6% vs. 22.0%). The residents with a bachelor’s degree or above had higher anxiety levels than those who had attended college or below (29.7% vs. 23.8%). Medical professionals were more likely to have anxiety than others (35.2% vs. 23.6%). The higher a resident’s income was, the more likely the respondent was to be anxious. The proportion of residents with anxiety was lower for those who communicated through online video many times a day than it was for those who communicated less frequently. The proportion of residents with anxiety symptoms was lower for those who frequently exercised than it was for those who did not (22.5% vs. 29.7%). In addition, there were significant differences in the depression level of residents based on place of residence, education, occupation, monthly income, frequency of online video communication, and exercise.

**Table 1:** The anxiety and depression of Wuhan residents in facing the epidemic of COVID-19.

Variable	*N* (%)	Anxiety	*χ*^2^	*P*	Depression	*χ*^2^	*P*
Gender							
Male	376 (30.3)	84 (22.4)	7.044	0.008	94 (25.3)	3.225	0.073
Female	866 (69.7)	256 (29.7)			261 (30.3)		
Age (years old)							
≤30	912 (73.4)	223 (24.5)	15.256	<0.001	253 (28.0)	1.070	0.301
>30	330 (26.6)	117 (35.8)			102 (31.0)		
Marital status							
Unmarried	824 (66.3)	186 (22.7)	28.445	<0.001	226 (27.6)	1.604	0.205
Married	418 (33.7)	154 (37.0)			129 (31.1)		
Residence area							
Rural	452 (36.4)	99 (22.0)	10.539	0.001	109 (24.2)	7.216	0.007
Urban	790 (63.6)	241 (30.6)			246 (31.4)		
Education							
College and below	390 (31.4)	93 (23.8)	4.554	0.033	90 (23.1)	10.653	0.001
Bachelor’s degree and above	852 (68.6)	253 (29.7)			274 (32.3)		
Occupation							
Others	788 (63.5)	186 (23.6)	19.413	0.000	208 (26.4)	8.821	0.003
Medical Staff	454 (36.5)	160 (35.2)			156 (34.4)		
Monthly income (CNY)							
<1000	554 (44.6)	114 (20.7)	24.509	<0.001	125 (22.7)	17.971	<0.001
1000–5000	389 (31.3)	121 (31.3)			132 (34.3)		
>5000	299 (24.1)	105 (35.2)			98 (32.9)		
Network communication							
No communication	309 (24.9)	68 (22.1)	6.574	0.037	61 (19.9)	17.155	<0.001
<2 times a day	438 (35.3)	122 (28.0)			130 (30.3)		
≥2 times a day	495 (39.9)	150 (30.4)			164 (33.3)		
Exercise							
Regular exercise	378 (30.4)	85 (22.5)	6.697	0.010	81 (21.4)	14.415	<0.001
No exercise	864 (69.6)	255 (29.7)			274 (32.0)		
Total	1242 (100.0)	346 (27.5)			364 (29.3)		

*COVID* Corona Virus Disease, *CNY* China Yuan.

The demographic characteristics of residents and the differences in their sleep quality and coping styles are shown in Table 2. Of the 1242 residents, 30.0% had sleep disorders, and 29.8% responded negatively to COVID-19. There were significant differences in residents’ sleep quality based on age, marital status, place of residence, monthly income, frequency of online video communication, and exercise. The proportion of residents with sleep disorders was lower for those ≤30 years than it was for those >30 years old (27.7% vs. 38.3%). The married residents reported a higher rate of sleep disorder than did the single respondents (36.7% vs. 27.4%). Urban residents were more likely to have sleep problems than were rural residents (34.3% vs. 23.9%). Respondents with bachelor’s degrees and above had a higher rate of sleep disorder than did those who had attended college or below (33.3% vs. 24.6%). Medical professionals reported a higher rate of sleep disorder than did others (36.6% vs. 27.0%). The higher the income, the more likely respondents were to suffer sleep problems. The proportion of residents with sleep disorders was higher for those who communicated through the internet many times a day than it was for those who did so less frequently. The proportion of residents with sleep disorders was lower for those who frequently exercised than it was for those who did not (22.0% vs. 34.3%). However, there were no significant differences in the coping styles of the residents based on their characteristics, except for their education and exercise levels. The proportion of residents who responded to COVID-19 actively was higher among those who frequently exercised than it was among those who did not (23.5% vs. 32.5%).

**Table 2:** The sleep quality and coping styles to stress of Wuhan residents in facing the epidemic of COVID-19.

Variable	*N* (%)	Sleep disorder	*χ*^2^	*P*	Passive coping style	*χ*^2^	*P*
Gender							
Male	376 (30.3)	103 (27.4)	2.517	0.113	117 (31.1)	0.454	0.501
Female	866 (69.7)	276 (31.9)			253 (29.2)		
Age (years old)							
≤30	912 (73.4)	253 (27.7)	12.702	<0.001	271 (29.7)	0.009	0.923
>30	330 (26.6)	126 (38.3)			99 (30.0)		
Marital status							
Unmarried	824 (66.3)	226 (27.4)	11.200	0.001	238 (28.9)	0.963	0.326
Married	418 (33.7)	153 (36.7)			132 (31.6)		
Residence area							
Rural	452 (36.4)	108 (23.9)	14.803	<0.001	148 (32.7)	2.962	0.085
Urban	790 (63.6)	271 (34.3)			222 (28.1)		
Education							
College and below	390 (31.4)	96 (24.6)	9.410	0.002	146 (37.4)	15.887	0.000
Bachelor’s degree and above	852 (68.6)	283 (33.3)			224 (26.3)		
Occupation							
Others	788 (63.5)	213 (27.0)	12.533	0.000	223 (28.3)	2.292	0.130
Medical Staff	454 (36.5)	166 (36.6)			147 (32.4)		
Monthly income (CNY)							
<1000	554 (44.6)	117 (21.1)	42.241	<0.001	167 (30.1)	1.972	0.373
1000–5000	389 (31.3)	152 (39.8)			123 (31.6)		
>5000	299 (24.1)	110 (36.9)			80 (26.8)		
Network communication							
No communication	309 (24.9)	72 (23.3)	10.165	0.006	108 (35.0)	5.258	0.072
<2 times a day	438 (35.3)	144 (32.9)			122 (27.9)		
≥2 times a day	495 (39.9)	163 (33.0)			140 (28.3)		
Exercise							
Regular exercise	378 (30.4)	83 (22.0)	18.873	<0.001	89 (23.5)	10.130	0.001
No exercise	864 (69.6)	296 (34.3)			281 (32.5)		
Total	1242 (100.0)	380 (30.6)			370 (29.8)		

*COVID* Corona Virus Disease, *CNY* China Yuan.

In the multivariate logistic regression analysis (Table 3), residents who are the female (*OR* = 1.62, 95% CI: 1.21–2.16, *P* = 0.001), have been married (*OR* = 1.75, 95% CI: 1.27–2.41, *P* = 0.001), with monthly income (CNY) more than 1000 (1000–5000: *OR* = 1.44, 95% CI: 1.03–2.01, *P* = 0.035; >5000: *OR* = 1.47, 95% CI: 1.16–2.07, *P* = 0.046), and with no physical exercise (*OR* = 1.45, 95% CI: 1.08–1.93, *P* = 0.013) were more likely to have anxiety. Monthly income (CNY) more than 1000 (1000–5000: *OR* = 1.83, 95% CI: 1.36–2.45, *P* = 0.000; >5000: *OR* = 1.45, 95% CI: 1.04–2.01, *P* = 0.027), having online communication (compared with no online communication, 0–2 times a day: *OR* = 1.47, 95% CI: 1.03–2.09, *P* = 0.012; ≥2 times a day: *OR* = 1.68, 95% CI: 1.19–2.38, *P* = 0.003), and with no physical exercise (*OR* = 1.71, 95% CI: 1.28–2.29, *P* = 0.000) showed higher risk of depression. In the Table 4, factors significantly associated with sleep disorder among residents included female gender (*OR* = 1.36, 95% CI: 1.03–1.79, *P* = 0.022), bachelor’s degree and above (*OR* = 1.40, 95% CI: 1.05–1.86, *P* = 0.001), with monthly income (CNY) more than 1000 (1000–5000: *OR* = 2.61, 95% CI: 1.94–3.50, *P* = 0.000; >5000: (*OR* = 2.14, 95% CI: 1.55–2.95, *P* = 0.000), with no physical exercise (*OR* = 1.85, 95% CI: 1.38–2.47, *P* = 0.000), living in the urban areas (*OR* = 0.75, 95% CI: 0.57–0.99, *P* = 0.039), bachelor’s degree and above (*OR* = 0.54, 95% CI: 0.41–0.70, *P* = 0.000), and no exercise (*OR* = 1.71, 95% CI: 1.29–2.27, *P* = 0.000) were independently associated with passive coping style.

**Table 3:** Multivariate logistic regression of anxiety and depression.

Variables	*β*	*SE*	*Wald*	*P*	*OR*	95% CI
Anxiety						
Constant	−1.896	0.197	92.446	0.000		
Female (Ref:Male)	0.480	0.149	10.397	0.001	1.62	1.21–2.16
Married (Ref:Unmarried)	0.560	0.163	11.819	0.001	1.75	1.27–2.41
Monthly income (CNY) 1000–5000 (Ref:Monthly income <1000)	0.362	0.172	4.438	0.035	1.44	1.03–2.01
Monthly income (CNY) > 5000 (Ref:Monthly income <1000)	0.373	0.189	4.715	0.046	1.47	1.16–2.07
No exercise (Ref:Regular exercise)	0.368	0.148	6.183	0.013	1.45	1.08–1.93
Depression						
Constant	−1.997	0.224	79.566	0.000		
Monthly income (CNY) 1000–5000 (Ref:Monthly income <1000)	0.603	0.15	16.082	0.000	1.83	1.36–2.45
Monthly income (CNY) > 5000 (Ref:Monthly income <1000)	0.368	0.166	4.891	0.027	1.45	1.04–2.01
Network communication <2 times a day (Ref:No communication)	0.384	0.181	4.528	0.033	1.47	1.03–2.09
Network communication ≥2 times a day (Ref:No communication)	0.520	0.177	8.682	0.003	1.68	1.19–2.38
No exercise (Ref:Regular exercise)	0.538	0.148	13.164	0.000	1.71	1.28–2.29

*SE* standard error, *OR* odds ratio, *CI* confidential interval, *Ref.* reference.

**Table 4:** Multivariate logistic regression of sleep disorder and passive coping style.

Variables	*β*	*SE*	*Wald*	*P*	*OR*	95% CI
Sleep disorder						
Constant	−2.233	0.208	114.76	0.000		
Female (Ref:Male)	0.305	0.142	4.597	0.032	1.36	1.03–1.79
Education (bachelor’s degree and above) (Ref:College and below)	0.334	0.145	5.268	0.022	1.40	1.05–1.86
Monthly income (CNY) 1000–5000 (Ref:Monthly income <1000)	0.958	0.15	40.578	0.000	2.61	1.94–3.50
Monthly income (CNY) > 5000 (Ref:Monthly income <1000)	0.760	0.165	21.301	0.000	2.14	1.55–2.95
No exercise (Ref:Regular exercise)	0.613	0.148	17.167	0.000	1.85	1.38–2.47
Passive coping style						
Constant	−0.672	0.18	13.898	0.000		
Urban (Ref:Rural)	−0.287	0.139	4.282	0.039	0.75	0.57–0.99
Education (bachelor’s degree and above) (Ref:College and below)	−0.624	0.138	20.545	0.000	0.54	0.41–0.70
No exercise (Ref:Regular exercise)	0.537	0.145	13.756	0.000	1.71	1.29–2.27

*SE* standard error, *OR* odds ratio, *CI* confidential interval, *Ref.* reference.

## Discussion

This cross-sectional study describes Wuhan residents’ current psychological status, sleep quality, and coping styles in response to the COVID-19 epidemic. More than one-fourth of the population suffered psychological problems, including anxiety and depression, and about one third had sleep disorders. These rates are similar to previous studies with participants around China^13^, which suggests that not only Wuhan residents but also other communities throughout China need urgent psychological interventions. In addition to establishing robust psychosocial and mental health support, healthcare centers and professional psychological counselors should meet the basic daily needs of patients with mental disorders and deliver positive information about epidemic prevention, in order to prevent the occurrence of psychological problems^14^.

Regarding coping styles, approximately 70.2% of residents have actively responded to the epidemic by participating in activities, talking with others about worries, and looking on the bright side. In comparison, 29.8% relied on passive coping styles, such as escapism, smoking, and depending on others. We expect these findings to inform the quality and efficacy of psychological, sleep, and coping interventions in this public health crisis by the Chinese government and authorities around the world.

This study found that the prevalence rate of psychological problems among respondents was higher than that of the whole population. A series of epidemiological studies have been conducted in Hubei to assess the latest neuropsychiatric conditions. According to the results, at least one mental illness among Hubei residents has a prevalence rate of more than 17.5%^15^. In addition, the prevalence of residents’ anxiety and depression in the COVID-19 epidemic was higher than it was during the severe acute respiratory syndrome (SARS) epidemic in 2003, when it ranged from 13% to −32 and 18% to −26%^16,17^. The panic caused by the high contagion rate of the novel coronavirus and residents being forced to quarantine at home may account for the difference. Entertainment activities have been restricted for a month, which may have depressed the residents’ mood. They can only learn about news from other parts of the world through the internet, and what they see is about the epidemic, which aggravates their psychological burden. In addition, some residents have to work to earn income. However, the extended holidays and long periods of isolation have slashed the opportunity to work, and the increasing gap can increase residents’ anxiety. The epidemic may have lasting effects on residents’ psychological health, and the risk of psychological disorders may increase over time. The improvement in health literacy, the touching stories about the fight against the epidemic, and the spirit of unity may have buffered the current occurrence of mental health problems. Timely, effective interventions can help prevent the further development of residents’ psychological problems. There was no statistical difference in the prevalence of depression (among residents of different genders, age groups, and marital statuses), sleep quality (among residents of different genders), and coping style to this crisis (among residents of different genders, age groups, marital statuses, residence regions, monthly income levels, and frequency of online communication). Equally attentive interventions to psychological problems, sleep disorders, and passive coping styles should be provided for all residents.

In the present study, rural residents had fewer symptoms of anxiety and depression, perhaps because rural areas are farther away from the center of the epidemic, leaving rural residents feeling relatively safe. Moreover, except for the decrease in activities, their lives were not significantly affected. In this epidemic, urban areas directly experienced the outbreak, and more infections concentrated in cities than did in the countryside. Coupled with the lower population density, infection rates have been relatively low in rural areas. However, access to information has been limited in rural areas, reducing residents’ panic to some extent.

Multivariate logistic regression analysis showed that females were at a higher risk of developing anxiety and sleep disorders, which was consistent with previous studies^18–20^. Compared with males, females are socialized to experience their emotions more strongly and have negative views of their health, which can cause them to become more anxious^21,22^. Furthermore, endocrine system functioning is also often implicated in biological explanations of sex differences in mental health outcomes. For example, a decline in estrogen might explain an increase in the risk of anxiety in women. High-intensity workloads have been identified as negatively influencing the neuroprotective effects of estrogen and other hormones^23^. The effective treatment of anxiety in women must address mental illness and include comprehensive interventions, such as hormone regulation.

In this study, most of the residents aged 30 years and older were married, with a sense of responsibility for their families. They tended to worry about their family members’ health instead of themselves, which could explain the higher prevalence of anxiety and sleep disorders among the married respondents^24^. Likewise, residents with a monthly income of more than 1000 CNY had a relatively higher risk of anxiety, depression, and sleep disorder. This group may have had a higher standard of living before the epidemic, which may have lessened their standard of living and inconvenienced them.

In the long term, an appropriate amount of physical exercise helps to increase the weight of the cerebral cortex. Physical exercise can promote blood circulation, provide sufficient oxygen for the movement of brain nerve cells to maintain adequate energy supply, improve the stability and flexibility of the central nervous system function, stimulate the brain, and help to prevent various psychological problems, such as anxiety, depression, and sleep disorders^25^. In addition, physical exercise can divert attention from the epidemic and reduce panic; thus, it is a positive coping mechanism in the face of the epidemic. A small number of studies have suggested that exercise is as effective as antidepressants for reducing symptoms of depression^26^. However, the attendance rates for exercise activities were already low in China, suggesting that health educators should publicize exercise’s positive effects on psychological disorders and encourage the public to do it regularly. During the epidemic, the public was asked to stay at home to protect vulnerable groups^27^. Residents have been communicating with the outside world through the internet, and online content has focused on the epidemic; mutual worry may have depressed people communicating frequently online.

Sleep quality is sensitive to psychological status^28^. In this study, approximately 30.6% of the residents showed sleep problems. In addition to the common influencing factors, such as gender, monthly income, and physical exercise, residents with a lower education level were more likely to suffer from sleep disorders. Since COVID-19 is a novel infectious disease with a high mortality rate, residents’ unfamiliarity has made them worry, which might have led to sleep problems. This effect could be more prominent in residents with lower education levels. Sleep neural pathways are closely connected and partly overlap with neural pathways regulating affect, cognition, and other important brain functions^29^. People with psychological disorders tend to worry, and they may focus on the adverse outcomes of this epidemic, making their brains remain in a state of tension, excitement, and alertness, and leading to insomnia and a vicious circle^30^. Individuals’ sleep quality and mental health status interact with each other. Sleep problems induce a poorer mental health status, and the effect of sleep problems on mood can be twice as large as mood’s effect on sleep^31^. Education level also positively influences a resident’s coping style. Generally, education level has been positively associated with higher awareness and compliance with the prevention and control of the COVID-19 epidemic. Therefore, during isolation or quarantine, residents with higher education levels may have adopted more proactive coping patterns, such as reading, physical activity, and seeking psychological support from family.

In addition, residents with less physical exercise were more likely to have sleep disorders and passive coping styles^32^. Physical exercise creates arousal and triggers the release of endorphins, noradrenaline, serotonin, and dopamine, which stimulate an individual’s spirit and can cause “exercise-induced euphoria”, which helps the exerciser feel peaceful, safe, and confident^33,34^. Physical exercise is an active coping style; thus, the function between sleep quality, coping style, and physical exercise may be bidirectional. In this epidemic, most residents were isolated in their homes, which limited their opportunity to exercise. About 69.6% of the residents in our study reported less physical exercise. Residents should find appropriate ways to exercise to improve their sleep quality and reactions to stress from the epidemic.

### Strengths and limitations

This timely survey of the psychological, sleep, and coping statuses of Hubei residents facing the COVID-19 epidemic included 1242 individuals, which is a larger sample size than that of most relevant studies. However, some limitations should be noted. First, the cross-sectional design of this study limited our ability to establish causal relationships among study variables. Second, this study was only conducted in Wuhan, which might limit its generalizability to other regions of China. However, Wuhan was the worst-affected city by the outbreak, and our findings on the residents of urban Wuhan might be the most representative.

## Conclusion

In facing the COVID-19 epidemic, Wuhan residents reported relatively poor psychological statuses and sleep quality, and their rate of passively coping with the stress was relatively higher. To fight the epidemic better, authorities must promote timely interventions to prevent residents’ mental health problems and increase residents’ confidence in responding positively to stressful events.
